# A Minimal Dose of Electrically Induced Muscle Activity Regulates Distinct Gene Signaling Pathways in Humans with Spinal Cord Injury

**DOI:** 10.1371/journal.pone.0115791

**Published:** 2014-12-22

**Authors:** Michael A. Petrie, Manish Suneja, Elizabeth Faidley, Richard K. Shields

**Affiliations:** 1 Department of Physical Therapy and Rehabilitation Science, Carver College of Medicine, The University of Iowa, Iowa City, Iowa, United States of America; 2 Department of Internal Medicine, Carver College of Medicine, The University of Iowa, Iowa City, Iowa, United States of America; 3 Department of Veterans Affairs, VA Medical Center, Iowa City, Iowa, United States of America; Rutgers-Robert wood Johnson Medical School, United States of America

## Abstract

Paralysis after a spinal cord injury (SCI) induces physiological adaptations that compromise the musculoskeletal and metabolic systems. Unlike non-SCI individuals, people with spinal cord injury experience minimal muscle activity which compromises optimal glucose utilization and metabolic control. Acute or chronic muscle activity, induced through electrical stimulation, may regulate key genes that enhance oxidative metabolism in paralyzed muscle. We investigated the short and long term effects of electrically induced exercise on mRNA expression of human paralyzed muscle. We developed an exercise dose that activated the muscle for only 0.6% of the day. The short term effects were assessed 3 hours after a single dose of exercise, while the long term effects were assessed after training 5 days per week for at least one year (adherence 81%). We found a single dose of exercise regulated 117 biological pathways as compared to 35 pathways after one year of training. A single dose of electrical stimulation increased the mRNA expression of transcriptional, translational, and enzyme regulators of metabolism important to shift muscle toward an oxidative phenotype (PGC-1α, NR4A3, IFRD1, ABRA, PDK4). However, chronic training increased the mRNA expression of specific metabolic pathway genes (BRP44, BRP44L, SDHB, ACADVL), mitochondrial fission and fusion genes (MFF, MFN1, MFN2), and slow muscle fiber genes (MYH6, MYH7, MYL3, MYL2). These findings support that a dose of electrical stimulation (∼10 minutes/day) regulates metabolic gene signaling pathways in human paralyzed muscle. Regulating these pathways early after SCI may contribute to reducing diabetes in people with longstanding paralysis from SCI.

## Introduction

Muscle paralysis after a spinal cord injury (SCI) triggers a cascade of events that disrupts the metabolic homeostasis of paralyzed muscle. Healthy skeletal muscle is involved with over 70% of daily glucose utilization [1]. Paralyzed muscle rapidly atrophies and transforms oxidative fibers into predominantly fast-twitch, glycolytic fibers [2]–[8]. Skeletal muscle that becomes glycolytic is a precursor to decreased insulin receptor sensitivity [9]. Individuals with SCI are at a higher risk of developing metabolic syndrome, diabetes, heart complications, and renal failure [10]–[14]. We have demonstrated that regular training of paralyzed muscle reduces muscle atrophy, preserves fatigue-resistance, and maintains the underlying skeletal system in people with SCI [6], [15]–[17]. However, we are not aware of the specific genes regulated by an acute bout of minimal muscle activity as compared to a long duration minimal muscle activity program in humans with paralysis.

Gene expression profiling is one method to survey the genome for mRNA transcripts common to a specific phenotype [18]. For example, through gene set enrichment methods, diabetes was linked to a decrease in the expression of oxidative phosphorylation signaling pathways in skeletal muscle [18]. People with paralysis experience limited muscle activity after the 10–30 minute bout of exercise using electrical stimulation, which is unlike people without paralysis, who are active between exercise bouts. We are interested in understanding if a single dose of muscle activity (muscle activated for less than.6% of the day equal to ∼10 minutes) versus a chronic dose of muscle activity (.6% of the day performed regularly over 1 year) regulates distinct gene transcription and metabolism pathways. No previous report, to our knowledge, has determined if acute and chronic muscle activity, induced electrically, regulates genes associated with glycolysis, tricarboxylic acid cycle (TCA), fatty acid oxidation, oxidative phosphorylation, and mitochondria dynamics (fission, fusion, and biogenesis) in people with SCI.

Key muscle transcription factors and co-activators are known to be responsive to metabolic and mechanical stress induced through muscle contractions in healthy people [19]. Previous studies have identified several of these stress response genes including peroxisome-proliferator-activated-receptor-gamma-coactivator-alpha (PGC-1α) [18], [20]–[22]; nuclear orphan receptor-1 (NOR-1/NR4A3) [23], [24]; interferon-related developmental regulator-1 (IFRD1) [25]; and actin-binding Rho-activating protein (ABRA/STARS) [21], [26], [27]. The induction or repression of these major transcription factors would trigger a cascade of events ultimately leading to the transformation of the underlying metabolic state of paralyzed skeletal muscle. Accordingly, genes associated with glycolysis (PDK4, PDHA1, PDHB, and PDHX) [28]–[33], fatty acid oxidation (ACADVL, ACADL, ACAD8, ACAD9) [33]–[36], tricarboxylic acid cycle (TCA) (BRP44, BRP44L, OGDH, SDHB) [33], [37], oxidative phosphorylation (NDUFB1, NDUFA2, CYC1, COQ10A) [33], [38], and mitochondria dynamics including fission, fusion, and biogenesis (MFF, OPA1, MFN1, and MFN2) [33], [39], [40] and their associated pathways are the primary focus of this investigation.

The purpose of this study is to determine the effects of a single dose and a chronic dose of electrical stimulation on human paralyzed muscle. We expect a single dose of muscle activity will result in an increased expression of metabolic transcription factors associated with the transformation of fibers from fast to slow. In contrast, we expect the chronically trained muscle will show the long term stable expression of genes associated with the maintenance of oxidative metabolic pathways, despite the minimal daily activity performed (10 minutes/day). Overall, these findings will support the hypothesis that a minimal dose of muscle activity, performed regularly, is an important and powerful metabolic regulator of skeletal muscle health, even in people with paralysis, who cannot exercise throughout the day.

## Methods

### Subjects

Five human subjects (30.40±4.39 years of age) with complete paraplegia were analyzed in this study. The protocol was approved by the University of Iowa Human Subjects Review Board, and all subjects provided written informed consent before participating. Three subjects received a single dose of electric muscle stimulation unilaterally 3 hours before a bilateral soleus muscle biopsy; whereas two subjects were muscle biopsied twice after training for >1 year (technical reference). All subjects had complete paraplegia (ASIA-A) at or below T4 and had been paralyzed for over 1.5 years. None of the subjects used electrical muscle stimulation of the soleus prior to the start of this study. The training subjects sustained over 81% compliance with the training protocol. The training subjects discontinued training 5 days prior to the soleus muscle biopsy procedure to capture the homeostatic effect of long term training and minimize the influence an acute dose of activity has on gene regulation. All biopsies were normalized to the same person's limb that did not receive training. The control limb has previously been shown to remain physiologically identical when the opposite limb is being trained [3], [4], [6], [15], [41], [42]; however, it is conceivable that some gene regulation may occur in the control limb from the training.

### Acute Electrical Stimulation Protocol

The acutely stimulated subjects had a single session of unilateral muscle stimulation of the soleus muscle. None of these individuals had received prior electrical stimulation. Subjects sat in their wheelchair while the experimenter positioned one leg into the testing apparatus in order to elicit an isometric contraction of the soleus muscle [3], [4], [6], [15], [41], [42]. The ankle and knee were flexed to 90° and the limb was secured to the apparatus with soft straps above the knee to provide resistance for an isometric contraction. The 90° knee angle minimizes the contribution of the gastrocnemius muscle to plantar flexion torque, creating a more reproducible measure of the performance of the soleus muscle [3], [4], [6], [41], [42].

Electrical stimulation was delivered to the plantar flexor muscles using reusable adhesive carbon electrodes. Stimulation was provided by a constant current electrical stimulator with a 0 to 400 milliamp range at 400 volts (Digitimer Model DS7A, Digitimer Ltd., Welwyn Garden City, Hertfordshire, England). The stimulator was controlled by digital pulses from a data-acquisition board (Metrabyte DAS 16F, Keithley Instruments Inc., Cleveland, Ohio, USA) housed in a microcomputer under custom software control. Single pulses were given at increasing intensity until maximal twitch torque was observed via an oscilloscope. Stimulation intensity was increased an additional 50% and remained at this level for the remainder of the experiment to insure supra maximal activation. Subjects received 5 warm-up contractions (10 Hz, 7 pulses per contraction) to potentiate the plantar flexor muscles and minimize the risk of muscle strain. After the warm-up contractions, the plantar flexor muscles were activated with 10 Hz stimulation trains (7 pulses) every 2100 ms for 120 isometric contractions. After a 5 minute rest, subjects received a second bout of 120 contractions at the same intensity and frequency. After the second bout, an experimenter used an indelible ink pen to mark the trained leg with the time that stimulation ended in preparation for the 3 hr post-activation bilateral soleus biopsy. The biopsy sampled fibers that had received the acute dose of electrical stimulation.

### Chronic Electrical Stimulation Protocol

The right soleus muscle was trained for at least one year then discontinued training 5 days prior to obtaining bilateral muscle biopsies. The training protocol consisted of 4 bouts of 125 contractions (15 Hz; train duration 667 ms) per day with a 5 minute rest period between each bout [6], [15]. The subjects trained one limb while the opposite limb did not receive any electrical stimulation training. The subjects performed a total of 333.3 seconds of supra maximal muscle activation per day, representing less than 0.6% of the day that the muscle was active.

### MRI Acquisition and Analysis

Due to the known association between metabolic risk and the fat to muscle ratio, we decided to illustrate the differences in phenotype between the trained and untrained limbs by using an axial-plane spin-echo, T1-weighted magnetic resonance (MR) image from the trained muscle and the untrained muscles of the calf and thigh (1.5 T Siemens Avanto Scanner). A three-dimensional gradient-echo-based sequence was used for high-resolution imaging. The acquisition parameters included repetition time (TR) of 15.0 ms and echo-time (TE) of 6.7 ms with a 512×256 matrix covering a field of view of 46 cm×18 cm and a 2.5-mm slice distance, creating a voxel volume of about 1.58 mm^3^. An MR was only obtained from subject 2, as subject 1 had metal fragments retained within his body from his initial injury [15]. The MR images were analyzed using a custom MATLAB^©^ (The Mathworks, Inc, Natick, MA, USA v.2011) script, which utilized image registration and segmentation algorithms implemented in the MATITK toolkit [43]. Using the image histogram, the images were segmented into three tissue types (adipose, muscle, and compact bone/background). After segmentation, the number of voxels corresponding to each tissue type was totaled and multiplied by the voxel volume of 1.58 mm^3^, providing an estimate to the total area for each tissue type within the limb. The trained and untrained limbs were separated into two regional sites (the distal thigh and the proximal calf) each consisting of 30 image slices. The proximal calf maximized the inclusion of the soleus muscle, while the distal thigh was used as a control to illustrate the specificity of training to the soleus muscle. The total muscle, fat, and background volumes were compared between the trained and untrained side. The total muscle and fat tissue content within each region was totaled and a ratio of muscle to fat volume was calculated.

### Muscle Biopsy and Exon Microarray Procedure

The biopsy procedure has been previously described [15]. Briefly, percutaneous muscle biopsies were taken from both the intervention soleus muscle and the control soleus muscle of each subject using a Temno biopsy needle (T1420, CardinalHealth) under ultrasound guidance within a sterile field. Both limbs were biopsied at the same time of day. Four passes of the needle were performed to obtain a wide sampling range within the muscle but limit injury to the patient for safety concerns. Following harvest, muscle biopsy samples were immediately placed In RNALater (Ambion) and stored at −80°C until further use. Total RNA was extracted using TRIzol solution (Invitrogen) according to the manufacturer's instructions, as described previously [15]. Microarray hybridizations were performed at the University of Iowa DNA Facility. Briefly, 50 ng of RNA was converted to SPIA amplified cDNA using the WT-Ovation Pico RNA Amplification System, v1 (NuGEN Technologies, San Carlos, CA, Cat. #3300) according to the manufacturer's recommended protocol. The amplified SPIA cDNA product was purified through a QIAGEN MinElute Reaction Cleanup column (QIAGEN Cat #28204) according to modifications from NuGEN. Four µg of SPIA amplified DNA were used to generate ST-cDNA using the WT-Ovation Exon Module v1 (NuGEN Technologies, Cat #2000) and again cleaned up with the Qiagen column as above. 5 µg of this product were fragmented (average fragment size  = 85 bases) and biotin labeled using the NuGEN FL-Ovation cDNA Biotin Module, v2 (NuGEN Technologies, Cat. #4200) per the manufacturer's recommended protocol. The resulting biotin-labeled cDNA was mixed with Affymetrix eukaryotic hybridization buffer (Affymetrix, Inc., Santa Clara, CA), placed onto Human Exon 1.0 ST arrays (Part No. 900650), and incubated at 45°C for 18 h with 60 rpm rotation in an Affymetrix Model 640 GeneChip Hybridization Oven. Following hybridization, the arrays were washed, stained with streptavidin-phycoerythrin (Molecular Probes, Inc., Eugene, OR), signal amplified with antistreptavidin antibody (Vector Laboratories, Inc., Burlingame, CA) using the Affymetrix Model 450 Fluidics Station. Arrays were scanned with the Affymetrix Model 3000 scanner with 7G upgrade and data were collected using the GeneChip operating software (GCOS) v1.4. All microarray data are MIAME compliant and have been submitted to the Gene Expression Omnibus (accession number pending).

### Exon Array Data Analysis

The Affymetrix Human Exon 1.0 ST arrays were normalized using a Robust Multi-array Average (RMA) and transformed into a log2 hybridization signal, reflecting the mean signal intensity of all exon probes specific for a particular mRNA transcript using Partek Genomic Suites (v6.6 Copyright © 2013 Partek Inc., St. Louis, MO, USA). The mRNA log2 hybridization signals were further analyzed using two techniques: a pathway analysis system and expression level profiling.

The pathway analysis was performed using the gene set enrichment analysis (GSEA) algorithm implemented in Partek Genomic Suites(v6.6 Copyright © 2013 Partek Inc., St. Louis, MO, USA) [44]. The Gene Ontology (GO) biological process database (version 3.1) was used to cluster differentially expressed gene transcripts to determine whether a particular pathway was likely up or down regulated in the acute and chronic groups. A false-detection rate (FDR) of 10%, lower than the recommended 25%, was used to determine those pathways significantly up or down regulated due to the small sample size.

The exon arrays were analyzed using an expression profiling technique, which compared the mRNA hybridization signals between the acute and chronic groups relative to each participant's control leg. MRNA with a log2 hybridization signal less than 2 standard deviations away from the mean hybridization signal were discarded from the dataset, restricting the analysis to mRNA transcripts with high signal intensity relative to background intensities. A fold-change (FC) was calculated for each gene transcript in the acute and chronic groups relative to the control limb. A gene transcript was considered to be differentially expressed, if 1) the FC was greater than 2.0 or less than 0.5 (indicating a 2.0 fold decrease in expression) and 2) the p-value for the gene was below 0.05 for an independent paired t-test comparing the stimulated and control. Due to the small sample size within each group, two muscles samples were independently extracted and hybridized for each subject, providing a technical replicate to minimize systematic errors.

### qPCR Procedure and Analysis

Muscle samples were homogenized in lysis buffer using a tissue homogenizer and hard tissue grinding tip (Omni). A column-based RNA extraction was subsequently performed using the RNEasy Fibrous Tissue Kit (Qiagen). DNAse was included in the protocol to ensure absence of genomic DNA in final samples. RNA samples were eluted in water and quantified via nanodrop method. In addition, the quality of each sample was assayed using the Agilent 2100 Bioanalyzer. High quality RNA samples were reverse transcribed using iScript supermix (Bio-Rad). Input quantity of RNA was standardized across all reactions (500 ng each). All cDNA samples were stored at −86°C. At the time of qPCR plate preparation, cDNA samples were diluted five-fold in water and analyzed via SYBR green technology using a custom PrimePCR plate (Bio-Rad). All samples were analyzed in duplicate at a final concentration of .5 ng/µL cDNA per 10 µL reaction using an ABI 7900 machine. Expression levels were converted to a relative fold change (FC) of the acutely stimulated or chronically trained limb to the control limb using the comparative C_Τ_ method described by Schmittgen and Livak [45]. Beta 2 macroglobulin (B2M) was used as the reference gene because it demonstrated a consistent expression across all limbs.

## Results

### Descriptive Analysis of Training Phenotype

**Figure 1:**
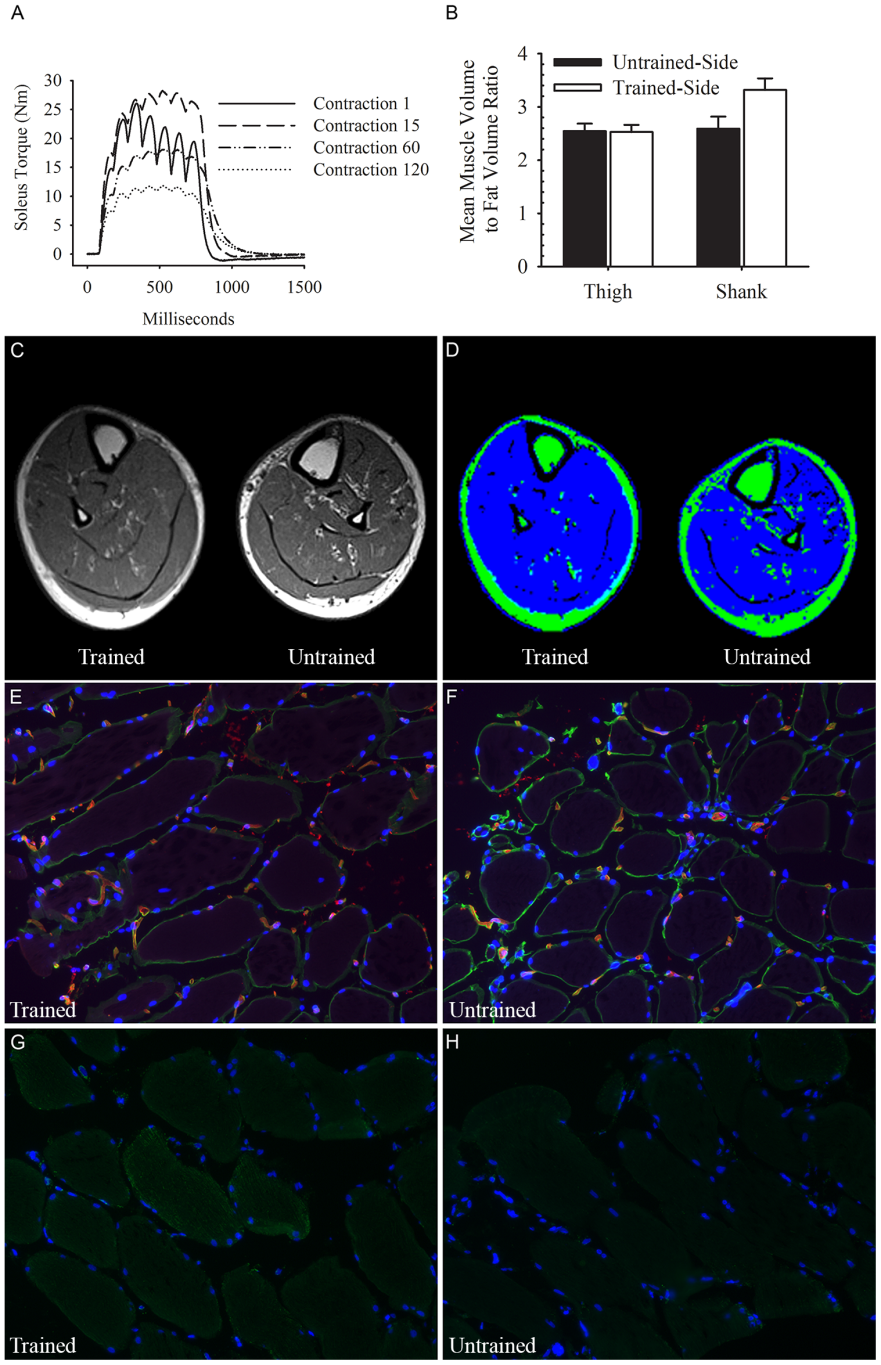
A representative example of the phenotype for a trained and untrained human paralyzed muscle. (A) A representative example of the torque produced during the stimulation of a chronically paralyzed human soleus muscle, contractions 1, 15, 60, and 120 during the first bout of electrical stimulation are illustrated. (B) The ratio of muscle to adipose tissue from several MR images slices of the proximal shank and distal thigh after >7 years of unilateral soleus electrical stimulation training in subject 1. A representative MR Image slice of the trained and untrained lower leg before (C) and after (D) implementing the muscle and fat tissue segmentation algorithm. Immunofluorescence stain for collagen IV (green) in a chronically trained (E) and untrained (F) paralyzed muscle. Note the loss of collagen IV (green) in the chronically trained muscle. Immunofluorescence stain (green) for mitochondrial distribution in a trained (G) and untrained (H) paralyzed muscle.

The chronically paralyzed muscle phenotype is highly fatigable, slows its contractile speed during repetitive activation, and potentiates early during electrical stimulation, findings that are consistent with fast fatigable glycolytic muscle (Fig. 1A). Long term training caused an increase in the lean muscle mass to adipose tissue ratio when compared to the untrained limb or to the upper thigh area which was not stimulated (Fig. 1B). The muscle hypertrophy following long term training and the reduced muscle fat are evident using MRI imaging from the trained and untrained limb of the same subject (Fig. 1C and 1D). Note the increased green marker within the atrophied untrained muscle indicating an increase of intramuscular fat. The chronic training phenotype also showed less collagen IV (Fig. 1E) and an increase in mitochondria signaling (Fig. 1G) when compared to the same subject's untrained control limb (Fig. 1F and 1H). The fatigue and contractile properties of the long term trained soleus muscle have been previously reported [6], [41], [42].

### Gene Enrichment Signaling Pathways

**Figure 2:**
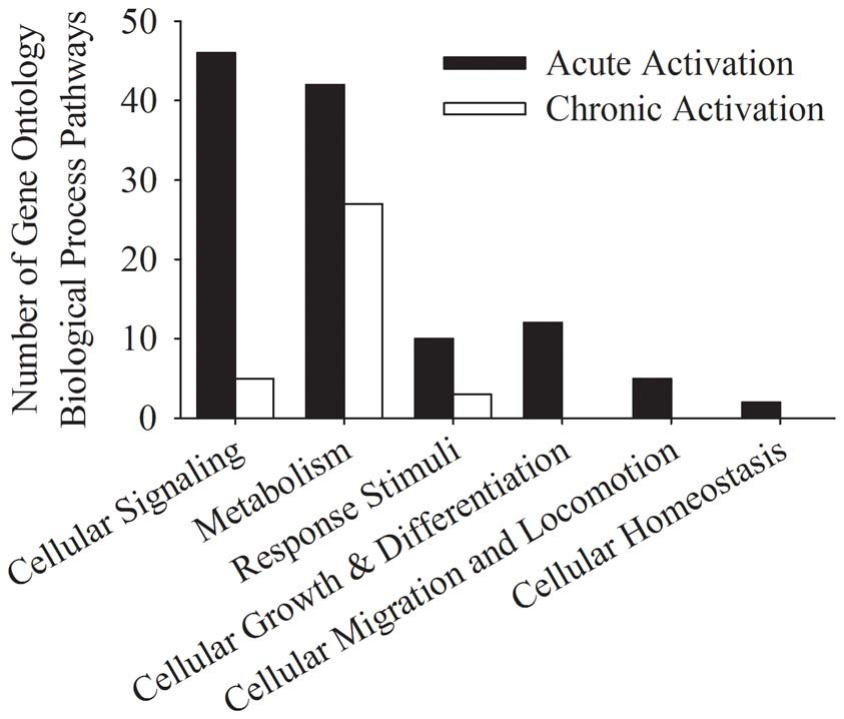
Gene Ontology(GO) Biological Process pathways upregulated by gene set enrichment analysis(GSEA) compared to non-stimulated limb. 117 pathways, primarily consisting of intra- and inter- cellular signaling pathways were upregulated 3-hours after a single session of exercise using electrical muscle stimulation (Acute Soleus Stimulation). 35 pathways, primarily consisting of metabolic pathways, were upregulated in subjects that trained >7 years using electrical muscle stimulation (Chronic Soleus Stimulation).

We measured over 16,000 mRNA transcripts from each muscle sample obtained. Using a conservative false discovery rate (FDR) of 10% and the gene ontology (GO) biological process database, we identified 117 and 35 pathways that were significantly up regulated after an acute dose and chronic dose of electrical stimulation, respectively (Fig. 2). Both the acute and chronic skeletal muscle groups demonstrated an increase in the expression of genes categorized as metabolic pathways; however, the genes with increased signal intensity differed between groups. An acute dose of electrical stimulation up regulated the expression of genes reported to be transcription factors and co-activators. In contrast, chronic training up regulated the expression of genes reported to be oxidative enzymes and metabolic transporter proteins. Many of the up regulated cell signaling and metabolic pathways involve the same genes (EIF4E, NR4A3, ABRA, EGR1, PGC-1Α, MYL3, MYH7, TNNT1, and ATP2A2). EIF4E, NR4A3, ABRA, EGR1, and PGC-1Α are transcription factors and co-activators important in maintaining oxidative muscle fibers. MYL3, MYH7, TNNT1, and ATP2A2 are contractile proteins observed in oxidative and not glycolytic muscle fibers.

### Individual Gene Analysis

Over 16,000 mRNA transcripts had a high hybridization signal in both the acute and chronic groups. Of those, 104 genes had a 2 fold increase in expression and 0 genes had a 2 fold decrease in expression after a single session of electrical muscle stimulation; however, after chronic stimulation training there were 66 genes with a 2 fold increase in expression, and 20 genes had a 2 fold decrease in expression. The top 10 genes with the highest relative fold change (Table 1 and 2) and lowest relative fold change (Table 3 and 4) are listed for the acute and chronic groups, respectively.

**Table 1:** Top 10 Expressed mRNA Gene Transcripts Following an Acute Dose of Electrical Muscle Stimulation.

Gene Symbol	Protein	Relative mRNA Level^a^
NR4A3	nuclear receptor subfamily 4 group A member 3	12.45±2.36
EGR1	early growth response protein 1	8.37±1.48
FOS	proto-oncogene c-Fos	7.98±3.43
GEM	GTP-binding protein GEM	6.59±1.17
KBTBD5	kelch-like protein 40	6.25±0.64
ABRA	actin-binding Rho-activating protein	5.98±0.4
IFRD1	interferon-related developmental regulator 1	5.71±0.73
CYR61	IGF-binding protein 10	5.70±1.46
PPARGC1A	peroxisome proliferator-activated receptor gamma coactivator 1-alpha	5.46±0.64
MYC	myc proto-oncogene protein	5.38±1.02

a mRNA expression levels reported as group mean ± standard deviation of fold-change relative to the control limb.

**Table 2:** Top 10 Expressed mRNA Gene Transcripts Following Chronic Training of Electrical Muscle Stimulation.

Gene Symbol	Protein	Relative mRNA Level^a^
MYH7	myosin heavy chain 7 (slow isoform)	11.69±4.93
MYL3	myosin light chain 3 (slow isoform)	9.07±3.75
PRUNE2	protein prune homolog 2	7.01±1.72
MYH6	myosin heavy chain 6 (cardiac muscle isoform)	6.76±2.5
PMS2CL	PMS2-C terminal-like protein	4.58±0.96
RSPO3	R-spondin-3	4.49±1.5
AGBL1	cytosolic carboxypeptidase 4	3.73±0.85
ENPP5	ectonucleotide pyrophosphatase/phosphodiesterase family member 5	3.33±0.36
MRAP2	melanocortin-2 receptor accessory protein 2	2.96±0.3
NPY6R	NPY6R neuropeptide Y receptor Y6	2.72±0.68

a mRNA expression levels reported as group mean ± standard deviation of fold-change relative to the control limb.

**Table 3:** Top 10 Repressed mRNA Gene Transcripts Following an Acute Dose of Electrical Muscle Stimulation.

Gene Symbol	Protein	Relative mRNA Level^a^
MSTN	growth/differentiation factor 8	0.56±0.06
ZNF30	zinc finger protein 30	0.61±0.04
FAM217B	protein FAM217B	0.62±0.08
YPEL2	protein yippee-like 2	0.62±0.03
ZNF429	zinc finger protein 429	0.63±0.07
POPDC2	popeye domain-containing protein 2	0.63±0.02
RAB30	ras-related protein Rab-30	0.63±0.05
TMEM242	transmembrane protein 242	0.64±0.16
RBM43	RNA-binding protein 43	0.64±0.07
ZNF92	zinc finger protein 92	0.64±0.06

a mRNA expression levels reported as group mean ± standard deviation of fold-change relative to the control limb.

**Table 4:** Top 10 Repressed mRNA Gene Transcripts Following Chronic Training of Electrical Muscle Stimulation.

Gene Symbol	Protein	Relative mRNA Level^a^
ACTN3	alpha-actinin-3	0.12±0.03
PVALB	parvalbumin alpha	0.26±0.19
MSTN	growth/differentiation factor 8	0.33±0.03
SH3RF2	putative E3 ubiquitin-protein ligase SH3RF2	0.36±0.09
HCN1	potassium/sodium hyperpolarization-activated gated channel 1	0.36±0.03
AQP4	aquaporin-4	0.37±0.04
SH2D1B	SH2 domain-containing protein 1B	0.39±0.09
MYLK2	myosin light chain kinase 2 (skeletal/cardiac muscle)	0.4±0.1
MYL5	myosin light chain 5	0.4±0.07
SLC22A3	solute carrier family 22 member 3	0.42±0.03

a mRNA expression levels reported as group mean ± standard deviation of fold-change relative to the control limb.

In the acute group, 8 of the top 10 high intensity transcripts function as transcription, translation, or enzymatic regulators (NR4A3, EGR1, FOS, GEM, ABRA, IFRD1, CYR61, and PGC-1α). PGC-1α, NR4A3, IFRD1, FOS, and ABRA trigger cellular processes needed to shift the muscle toward an oxidative phenotype, while CYR61 aids in initiating angiogenesis in muscle. PGC-1α, NR4A3, EGR1, IFRD1, FOS, and ABRA were over 2 fold higher following an acute bout of electrical stimulation in paralyzed muscle. In contrast, these genes were minimally altered in chronically trained paralyzed muscle. In the chronic group, 5 out of the 10 high intensity transcripts function as metabolic enzymes, protein transporters, and oxidative muscle proteins (MYH6, MYH7, MYL3, MYL2, and AGBL1). MYH6, MYL3, and MYH7 were at least 3 fold higher in chronically trained muscle relative to acutely stimulated paralyzed soleus muscle. Importantly, chronic training decreased genes associated with fast twitch muscle (ACTN3, MYLK2, and MYL5) and atrophy (MSTN) (Table 4). An acute session of electrical stimulation triggered a 1.8 fold decrease of MSTN (Table 3), and was the only common gene substantially decreased in both the acute and chronic groups.

### Transcription Factors and Muscle Fiber Types

**Figure 3:**
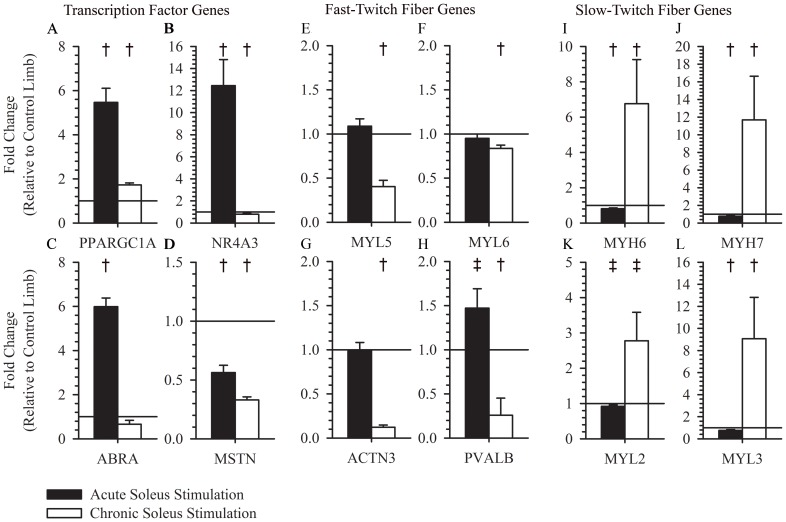
Expression of transcription factor, fast-twitch fiber, and slow-twitch fiber genes following acute or chronic stimulation. PGC-1α was increased 3 hours after a dose of muscle stimulation (5.46±0.64, p<0.001) and after >1 year of muscle training (1.73±0.09, p<0.002) (A). NR4A3 was increased 3 hours after a dose of muscle stimulation (12.45±2.36, p<0.001), while it was decreased after >1 year of muscle training (0.79±0.06, p = 0.046) (B). ABRA was increased after a single dose of muscle stimulation (5.98±0.40, p<0.001), but was unchanged after >1 year of soleus training (0.66±0.18, p<0.16) (C). MSTN was decreased 3 hours after a dose of muscle stimulation (0.56±0.06, p = 0.002) and after >1 year of muscle training (0.33±0.03, p<0.001) (D). MYL5 (0.040±0.07, p = 0.013), MYL6 (0.84±0.038, p = 0.030), and ACTN3 (0.12±0.025, p = 0.003) were all decreased after >1 year of muscle training (E, F, and G). There was no difference detected 3 hours after a dose of muscle stimulation for MYL5 (1.09±0.083, p = 0.45). MYL6 (0.95±0.048, p = 0.32), and ACTN3 (0.99,0.095, p = 0.72) (E, F, and G). PVALB was increased after a single dose of muscle stimulation (1.47±0.22, p = 0.074), but was decreased after >1 year of muscle training (0.26±0.19, p = 0.047) (H). MYH6 (6.76±2.50, p = 0.030), MYH7 (11.69±4.93, p = 0.025), MYL2 (2.78±0.80, p = 0.063), and MYL3 (9.07±3.75, p = 0.046) were increased after >1 year of muscle training, while they were decreased 3 hours after single session of muscle stimulation (0.81±0.04, p = 0.0073, 0.77±0.073, p = 0.030, 0.92±0.036, p = 0.066, 0.76±0.078, p = 0.037; respectively) (I, J, K, and L). † indicates a p-value <0.05 for a within group paired t-test. ‡ indicates a p-value <0.10 for a within group paired t-test.

After a preliminary analysis, we looked at the influence of acute and chronic soleus stimulation on a subset of genes linked to skeletal muscle phenotypes or metabolic capacity, specifically targeting genes linked to skeletal muscle transcription factors, fast-twitch muscle proteins, slow-twitch muscle proteins, glycolysis enzymes, fatty acid oxidation enzymes, tricarboxylic acid cycle enzymes, oxidative phosphorylation enzymes, and mitochondrial fission and fusion proteins. PGC-1α(5.46±0.64, p<0.001), NR4A3(12.45±2.36, p<0.001), and ABRA(5.98±0.40, p<0.001) are early response genes that were up regulated relative to the non-stimulated limb 3 hours after a single bout of electrical muscle stimulation. (Fig. 3A–C) However, after chronic electrical stimulation training only PGC-1α (1.73±0.09, p<0.002) was increased (Fig. 3A). NR4A3 and ABRA demonstrated a relative decrease with chronic electrical muscle stimulation training (Fig. 3B and C). MSTN was decreased both 3 hours after an electrical muscle stimulation exercise (0.56±0.06, p = 0.002) and chronic training (0.33±0.03, p<0.001) (Fig. 3D). Genes linked to fast-twitch muscle fibers were decreased after chronic electrical stimulation training, but were, in general, unaltered 3-hours after a single session of electrical muscle stimulation in chronically untrained paralyzed muscle (Fig. 3E–H). In contrast, genes linked to slow-twitch muscle fibers were increased in chronically trained muscle, but decreased 3 hours after a single session of electrical muscle stimulation (Fig. 3I–L).

### Glycolysis and Fatty Acid Oxidation

**Figure 4:**
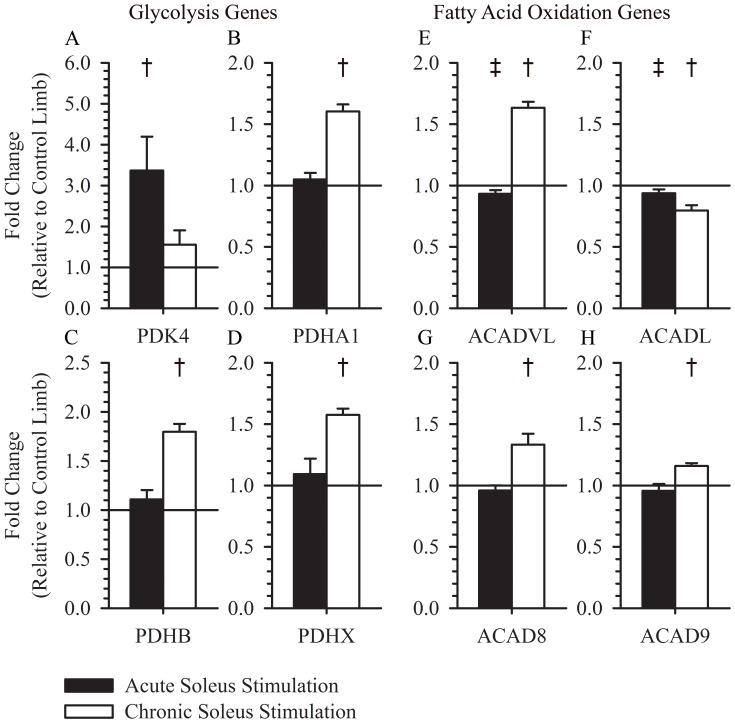
Expression of glycolysis and fatty acid oxidation genes following acute or chronic stimulation. PDK4 was increased 3 hours after a single session of muscle stimulation (3.37±0.83, p = 0.008), but was unchanged after >1 year of soleus training (1.55±0.35, p = 0.21) (A). PDHA1 (1.60±0.057, p<0.001) PDHB (1.80±0.08, p<0.001), and PDHX (1.57±0.05, p<0.001) were increased after >1 year of muscle training, but were unchanged 3 hours after a single session of muscle stimulation (1.05±0.05, p = 0.46, 1.11±0.09, p = 0.35, 1.09±0.13, p = 0.59; respectively) (B, C, and D). ACADVL (0.93±0.03, p = 0.064) was decreased 3 hours after a single session of muscle stimulation, but was increased after >1 year of muscle training (1.63±0.049, p = 0.049) (E). ACADL (0.94±0.031, p = 0.098) was decreased 3 hours after a single session of muscle stimulation and after >1 year of muscle training (0.80 and ACADL (0.94±0.031, p = 0.098) were decreased 3 hours after a single session of muscle stimulation 0.044, p = 0.025) (F). ACAD8 (1.33±0.089, p = 0.023) and ACAD9 (1.16±0.023, p = 0.006) were increased after >1 year of muscle training, but were unchanged 3 hours after a single dose of muscle stimulation (0.96±0.042, p = 0.33, 0.96±0.06, p = 0.39; respectively) (G and H). † indicates a p-value <0.05 for a within group paired t-test. ‡ indicates a p-value <0.10 for a within group paired t-test.

Glycolysis and fatty acid oxidation are the 2 primary pathways used to metabolize macromolecules in skeletal muscle. PDK4 was increased relative to the control limb 3 hours after a single session of electrical muscle stimulation training (3.37±0.83, p = 0.008), but was not increased with chronic muscle training (1.55±0.35, p = 0.21) (Fig. 4A). PDHA1(1.60±0.057, p<0.001) PDHB(1.80±0.08, p<0.001), and PDHX(1.57±0.05, p<0.001) were all increased after chronic electrical muscle stimulation training, but were not altered 3 hours after a single session of electrical muscle stimulation (1.05±0.05, p = 0.46, 1.11±0.09, p = 0.35, 1.09±0.13, p = 0.59; respectively). ACADVL(1.63±0.049, p = 0.049), ACAD8(1.33±0.089, p = 0.023) and ACAD9(1.16±0.023, p = 0.006) were increased after chronic electrical muscle stimulation training, but were unchanged or decreased 3 hours after a single session of electrical muscle stimulation (Fig. 4E,G, and H). ACADL was decreased after acute and chronic muscle training (0.94±0.031, p = 0.098, 0.80±0.044, p = 0.025, respectively), with a larger effect observed after chronic electrical muscle stimulation training (Fig. 4F).

### Tricarboxylic Acid Cycle, Oxidative Phosphorylation, and Mitochondrial Fission/Fusion

**Figure 5:**
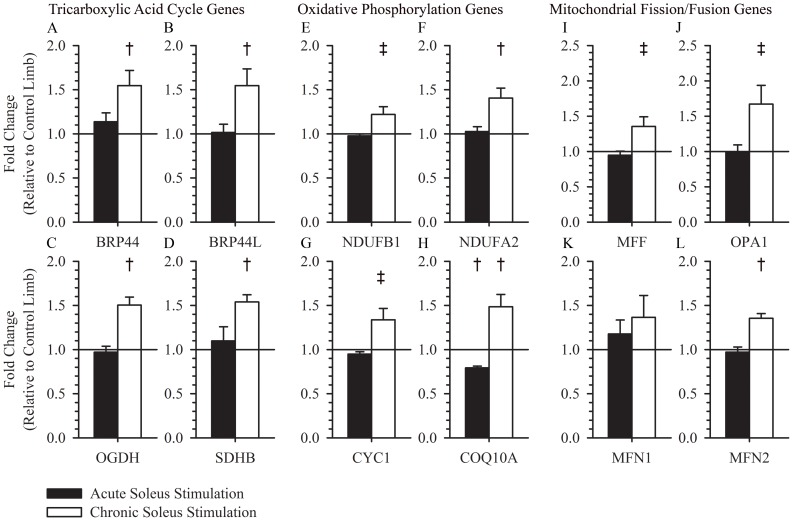
Expression of tricarboxylic acid cycle, oxidative phosphorylation, and mitochondrial fission/fusion genes following acute or chronic stimulation. BRP44 (1.55±0.17, p = 0.034), BRP44L (1.55±0.19, p = 0.036), OGDH (1.50±0.092, p = 0.007), and SDHB (1.54±0.081, p = 0.004) were increased after >1 year of muscle training, but were unchanged 3 hours after a single dose of muscle stimulation (1.14±0.099, p = 0.25, 1.02±0.093, p = 0.97, 0.97±0.067, p = 0.58, 1.10±0.16, p = 0.74; respectively) (A, B, C, and D). NDUFB1 (1.22±0.088, p = 0.067), NDUFA2 (1.40±0.11, p = 0.022), and CYC1 (1.34±0.13, p = 0.066) were increased after >1 year of muscle training, but were unchanged 3 hours after a single dose of muscle stimulation (0.98±0.02, p = 0.28, 1.03±0.05, p = 0.72, 0.95±0.03, p = 0.10; respectively) (E, F, and G). COQ10A was increased after >1 year of muscle training (1.49±0.14, p = 0.024), but was decreased 3 hours after a single dose of muscle stimulation (0.79±0.021, p<0.001) (H). MFF (1.35±0.14, p = 0.062), OPA (1.67±0.27, p = 0.074), and MFN2 (1.35±0.053, p = 0.004) were increased after >1 year of muscle training, but were unchanged 3 hours after a single dose of muscle stimulation (0.95±0.31, p = 0.31, 1.00±0.78, p = 0.77, 0.97±0.54, p = 0.54); respectively) (I, J, and L). MFN1 was unchanged after >1 year of muscle training (1.36±0.25, p = 0.22) and 3 hours after a single dose of muscle stimulation (1.18±0.16, p = 0.42) (K). † indicates a p-value <0.05 for a within group paired t-test. ‡ indicates a p-value <0.10 for a within group paired t-test.

The common substrate, acetyl CoA, following aerobic glycolysis or fatty acid oxidation is further oxidized within mitochondria via the tricarboxylic acid cycle and oxidative phosphorylation pathways. Genes responsible for monocarboxylic acid transport into the mitochondria (BRP44(1.55±0.17, p = 0.034) and BRP44L (1.55±0.19, p = 0.036)) were increased after chronic electrical muscle stimulation training and unchanged 3 hours after a single session of electrical muscle stimulation (Fig. 5A, B). A subset of rate limiting oxidative enzymes in the tricarboxylic acid (OGDH (1.50±0.092, p = 0.007) and SDHB (1.54±0.081, p = 0.004)) and oxidative phosphorylation (NDUFB1 (1.22±0.088, p = 0.067), NDUFA2 (1.40±0.11, p = 0.022), and CYC1 (1.34±0.13, p = 0.066), COQ10A (1.49±0.14, p = 0.024)) pathways were increased after chronic training but unchanged 3 hours after a single session of electrical muscle stimulation (Fig. 5C–H). MFF (1.35±0.14, p = 0.062), OPA (1.67±0.27, p = 0.074), MFN1 (1.36±0.25, p = 0.22), and MFN2 (1.35±0.053, p = 0.004) aid in regulating mitochondrial fission and fusion, and were increased with chronic training (Fig. 5I–L).

### qPCR Validation

**Figure 6:**
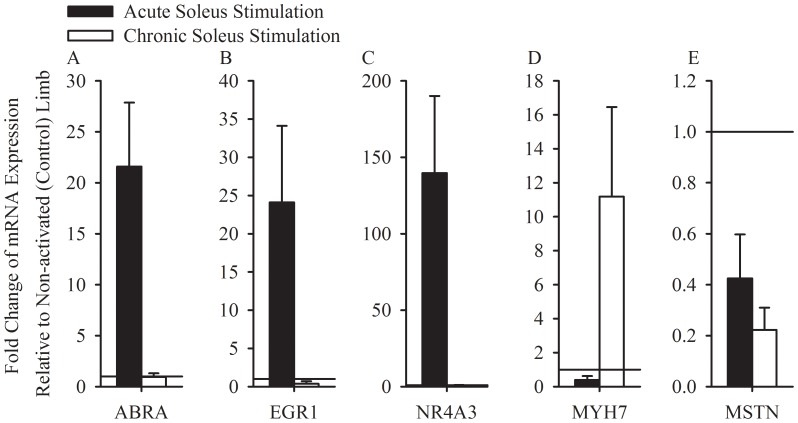
Confirmatory qPCR of a subset of genes important for metabolic and hypertrophy pathways in muscle. qPCR analysis of mean ABRA (A), EGR1(B), NR4A3(C), MYH7(D), and MSTN(E) levels for acutely stimulated muscles (black) and chronically trained muscles (white). (A–E) Data represented as mean ± standard error.

We verified the expression levels of five genes (ABRA, EGR1, MYH7, NR4A3, and MSTN) using qPCR. Similar to the exon microarray results, ABRA, EGR1, and NR4A3 expression was increased following an acute dose of electrical stimulation but was decreased or unchanged in the chronically trained muscle (Fig. 6 A,B, and C). Chronically training muscle resulted in a sustained increase of MYH7, while a single dose of activity minimally altered MYH7 expression (Fig. 6D). Chronic and acute muscle stimulation resulted in repression of MSTN, with a larger effect being seen within the chronically trained muscle (Fig. 6E).

## Discussion

We examined the mRNA expression response after a single session of exercise with electrical stimulation in paralyzed soleus muscle and compared that to the mRNA expression profile of the chronically trained paralyzed soleus muscle. The objective of this study was to advance our understanding of the influence of acute or chronic exercise on regulating gene expression important for metabolic health of skeletal muscle in humans with paralysis. The major findings of this study are 1) acute stimulation of paralyzed muscle regulates over 100 biological pathways as compared to less than 30 in chronically trained muscle; 2) acute electrically induced exercise up regulates transcriptional, translational, and enzyme regulators of metabolic pathways that shift muscle toward an oxidative phenotype (PGC-1α, NR4A3, IFRD1, ABRA, PDK4); 3) long term electrically induced exercise increased the chronic expression of glycolysis (PDHA1, PDHB, PDHX), fatty acid oxidation (ACADVL, ACAD8, ACAD9), tricarboxylic acid (BRP44, BRP44L, OGDH, SDHB), and oxidative genes (NDUFB1, NDUFA2, CYC1, COQ10A); 4) long term electrically induced exercise increased oxidative muscle fiber (MYH6, MYH7 MYL3, and MYL2) and mitochondrial fission/fusion (MFF, OA1, MFN1, MFN2) genes, but repressed glycolytic muscle (ACTN3, MYLK2, and MYL5) and muscle atrophy (MSTN) genes. Taken together, these findings support that the minimal dose (0.6% of muscle activity per day) of electrically induced exercise used in this study was sufficient to initiate and maintain several metabolic pathways in human paralyzed muscle. Whether preserving the metabolic capacity of skeletal muscle in humans after SCI improves systemic metabolic health remains unknown.

In 1995, we reported that the acutely paralyzed soleus muscle in humans is fatigue resistant and oxidative, but transforms into a highly fatigable, glycolytic muscle after 1 year of paralysis [4]. Subsequent long term training intervention studies verified that there was a feasible dose of daily muscle exercise, induced by electrical stimulation, that could sustain bone mineral density of the underlying skeletal system [6], [46], [47] and the phenotype of the soleus muscle including its size, fatigue resistance, muscle oxidative enzymes, reduced post activation potentiation, and reduced muscle contractile speeds [5], [6], [15], [41], [42]. The high incidence of metabolic disease (diabetes) in individuals with SCI [10]–[12], prompted us to question whether the transformation of paralyzed muscle to the glycolytic state contributes to the development of systemic metabolic syndromes consistent with skeletal muscle of people with diabetes [9]. We discovered, in this study, that a moderate dose of acute and chronic electrical stimulation regulates common and distinct genes and pathways involved with glucose metabolism.

This study demonstrated that a single dose of electrically induced exercise stressed muscle and triggered the transcription of genes important for controlling metabolic pathways. Further, chronic training of paralyzed muscle resulted in a consistent increase of genes and proteins important for an oxidative, slow-twitch phenotype. It is noteworthy that skeletal muscle adaptation requires a repetitive and long term (over a 1 year time) delivery of a stressor in order to up regulate the full complement of oxidative enzymes necessary to improve muscle endurance [6] and reduce muscle fat [15], [41] (see Fig. 1). The minimal dose needed to prevent skeletal muscle transformation after paralysis is not known. The magnitude of the gene regulation after acute and chronic electrical stimulation in this study is striking considering the muscle was active for less than 0.6% of the day, while the remainder of the day the muscle experiences little contractile stress.

Two factors likely contributed to the magnitude of gene signaling based on the design of this protocol. First, the frequency of stimulation was set to create an unfused tetany, which generated ∼50% of the maximal muscle force generating capacity, under isometric conditions. We chose this level of stimulation because we believed 50% of maximal force mechanically strains tissues that modulate muscle signaling and we wished to limit the total force of the muscle to a level that would not induce injury to the underlying skeletal and soft-tissues. Second, we supra maximally activated the muscle, which assured all fibers of the soleus muscle were activated. This level of muscle activity is unique and specific to electrical stimulation induced exercise. For example, this dose of exercise cannot be duplicated in humans under volitional control, as motor unit recruitment, via the size principle [48], and rate coding [49] will not allow a volitionally driven muscle to be fully activated at a low firing frequency. Because electrical stimulation exercise recruits motor units differently from volitional exercise, it is risky to assume that training principles learned from normal volitionally driven exercise apply to paralyzed muscle exercised through electrical stimulation.

The unique method of stimulating paralyzed muscle induced the peroxisome-proliferator-activated-receptor-gamma-coactivator-alpha (PGC-1α) gene. Increased PGC-1α expression results in muscle hypertrophy, oxidative, and slow-twitch protein expression, while decreased levels lead to the conversion to glycolytic proteins and a transformation to a faster-twitch, less oxidative muscle fiber [20], [21], [50]. In able-bodied individuals, bouts of exercise have been shown to increase the expression of PGC-1α, with large increases seen when the muscle has been metabolically challenged [44]. In this study, a single dose of electrical stimulation challenged the predominantly fast, glycolytic paralyzed muscle resulting in over a 5 fold increase in PGC-1α expression. Interestingly, the chronically trained soleus muscle demonstrated a sustained increase in PGC-1α expression when compared to the opposite untrained paralyzed muscle. However, the PGC-1α expression was far less than that induced just 3 hours after a single dose of electrical stimulation. The elevated PGC-1α expression in the chronically trained state suggests that muscle protein adaptations, induced by the repetitive challenge through chronic training, now prevents the buildup of metabolites (reactive oxygen species) that are known to initiate the cascade of events that follow PGC-1α transcription [51].

The nuclear orphan receptor-1 (also referred to as NR4A3) assists in regulating muscle metabolic phenotypes, with increased expression resulting in a more oxidative muscle fiber [23], [24]. Several environmental stressors have been shown to regulate NR4A3 expression including β-adrenoreceptors, cold, fatty acids, glucose, insulin, cholesterol, melanocortin, and thiazolidinediones [23], [24]. However, our single dose of exercise, induced by electrical stimulation, caused a>5 fold increase in NR4A3 mRNA 3 hours after exercise. Although, this study did not investigate the specific parameters of electrical stimulation that optimize the expression of NR4A3, we know that long term use of this stimulation ultimately leads to less than a 1 fold increase in NR4A3 mRNA expression, suggesting that NR4A3 plays a role in initiating a metabolic shift, but may be less important during periods of inactivity.

Actin-binding Rho-activating protein (ABRA) has also been shown to be instrumental in triggering the transformation of muscle to a more oxidative muscle fiber phenotype through the activation of the PGC-1α pathway [21], [26], [27]. ABRA is located within the sarcomere and is thought to be a critical link in triggering cellular adaptations by transducing the mechanical stress to intracellular communication pathways [26], [52]. Additionally, ABRA has been shown to translocate to the nucleus and may act as a transcriptional co-activator or transcription factor [21], [52]. Interestingly, ABRA expression has been show to decrease with immobilization and during disuse atrophy [26], [53] and seems to be increased in diabetic muscle [26], [54].

We limited our analysis to genes with a large differential expression (>2 FC) compared to the untrained limb. However, limiting to a>2 FC eliminates transcripts that are differentially expressed but to a lesser magnitude. We explored genes that did not meet the 2 FC thresholds, but have important roles in regulating oxidative metabolism. BRP44 and BRP44L are two genes that control the transport of pyruvate from the cytosol to the mitochondria for aerobic oxidation [55]. Interestingly, BRP44 and BRP44L were not altered 3 hours after exercise induced by electrical stimulation. However, with chronic training, both increased significantly with a>1.5 FC compared to the untrained limb.

The mitochondrion is a critical organelle for skeletal muscle metabolism, generating over 90% of a cell's energy [56]. The mitochondrial stress response contributes to the development of insulin resistance [57]. In skeletal muscle, mitochondria form a complex network that may be an indicator of the metabolic health of a muscle fiber. Mitochondria are regulated by the balance between mitochondrial fission and fusion [56]. The lack of mitochondrial fusion and excessive mitochondrial fission contributes to mitochondrial dysfunction, decreasing the oxidative capacity of the muscle fiber [56]. Chronic muscle training, using electrical muscle stimulation, increased the expression of mitochondrial fusion genes. However, mitochondrial fission genes (DNM1L and INF2) [39] were unchanged (data not shown). Interestingly, the mitochondrial fission and fusion genes were minimally altered 3 hours after a single dose of muscle activity. However, a single dose of muscle activity significantly increased the expression of PGC-1α, which is a key initiator of mitochondrial biogenesis. Taken together, repeated long-term exposure to the stress of muscle activity is important to cause cellular adaptations needed to alter the oxidative capacity of a muscle.

The findings support that exercise, through electrical stimulation, is a powerful method to stress skeletal muscle. Exercised skeletal muscle has a high oxidative capacity, and can more rigorously respond to insulin and metabolize glucose [22], [58]. The change in muscle insulin receptor sensitivity associated with routine exercise may, in part, explain several of the pathways regulated in this study [51], [59]–[62]. For individuals with SCI, an ability to sustain the size and oxidative capacity of skeletal muscle may be paramount to improving overall metabolic health.

Future studies are underway to deliver interventions shortly after the injury that efficiently regulates skeletal muscle with the goal to optimize the health of people with paralysis. The optimal electrical stimulation dose (frequency, current intensity, duration, work-rest) to most efficiently/effectively elicit long term cellular adaptations of paralyzed muscle are not known. By targeting specific gene signaling pathways regulated by exercise, future studies may develop “scientifically grounded” electrical stimulation interventions before initiating expensive and long-term training programs to restore the metabolic capacity of paralyzed skeletal muscle. Future studies examining various doses of electrical stimulation may selectively regulate quite different gene signaling pathways and be prescribed based on the needs of the individual with SCI (hypertrophy vs. metabolism; acute vs. chronic).

### Methodological and Clinical Considerations

The results of this study provide a “snapshot” into the effects that a defined dose of muscle activity has on gene regulation in human paralyzed muscle. We used an intervention to sufficiently “stress” the paralyzed muscle while also not injuring the underlying muscle or skeletal structures. In addition, we used a biopsy technique that minimized the amount of tissue harvested in order to reduce risk of secondary complications associated with venous stasis in humans with paralysis. We required nearly all of the harvested tissues to sufficiently extract enough RNA to examine cell signaling pathways, which was the primary objective of this study. However, this precluded us from carrying out comprehensive proteomic studies and microscopy studies. Indeed, we do not know how long gene expression was altered after 3 hours or the dose needed to induce an actual change in protein. We do know that chronic training of ∼10 minutes of activity/day caused a significant change in the functioning phenotype (fatigue, torque, underlying bone tissue) and a stable up regulation of several metabolic signaling pathways. While the number of participants in this study was not large, each subject had a control limb serving as a within subject comparison, constituting the identical genotype to contrast the effects of the electrical stimulation intervention. In addition, the long term trained subjects, while limited in number, represent a novel example of the capacity for muscle tissue to adapt when exposed to a regular (>1 year) but a modest dose of electrically induced exercise (0.6%/day).

## Conclusions

A modest dose of acute or chronic electrical stimulation of paralyzed muscle regulated distinct biological pathways. Taken together, these findings support that acute and chronic electrical stimulation intervention (∼10 minutes/day) regulates key metabolic gene signaling pathways. Early regulation of these pathways after SCI may assist in preventing diabetes in people with SCI.
